# Tuberculosis Treatment Completion in a United States/Mexico Binational Context

**DOI:** 10.3389/fpubh.2017.00118

**Published:** 2017-05-24

**Authors:** Celina I. Valencia, Kacey Ernst, Cecilia Ballesteros Rosales

**Affiliations:** ^1^Department of Community, Environment, and Policy, Mel and Enid Zuckerman College of Public Health, University of Arizona, Tucson, AZ, USA; ^2^Department of Epidemiology and Biostatistics, Mel and Enid Zuckerman College of Public Health, University of Arizona, Tucson, AZ, USA; ^3^Division of Public Health Practice and Translational Research, Mel and Enid Zuckerman College of Public Health, University of Arizona, Tucson, AZ, USA

**Keywords:** U.S./Mexico border, tuberculosis, binational health, economic burden, mobility

## Abstract

**Background:**

Tuberculosis (TB) remains a salient public health issue along the U.S./Mexico border. This study seeks to identify the social and structural factors, which are associated with TB disease burden in the binational geographic region. Identification of barriers of treatment completion provides the necessary framework for developing evidence-based interventions that are culturally relevant and context specific for the U.S./Mexico border region.

**Methods:**

Retrospective study of data extracted from medical charts (*n* = 439) from Yuma County Health Department (YCHD) (*n* = 160) and Centro de Salud San Luis Río Colorado (*n* = 279). Patients currently accessing TB treatment at either facility were excluded from the study. Chi-square, unadjusted odds ratios, and logistic regression were utilized to identify characteristics associated with successful TB treatment in this population.

**Findings:**

The study population was predominantly male (*n* = 327). Females were more likely to complete TB treatment (OR = 3.71). The absence of drug use and/or the absence of an HIV positive diagnosis were found to be predictors of TB treatment completion across both clinical sites. Forty-four percent (43.59%) (*n* = 85) TB patients treated at CDS San Luis did not complete treatment versus 40.35% (*n* = 49) of TB patients who did not complete treatment at YCHD. Moving from the area or being deported was the highest category (20.78%) for incomplete TB treatment in the population (*n* = 64) across both clinical sites.

## Introduction

The World Health Organization (WHO) classifies the tuberculosis (TB) epidemic as one of the most pressing public health issues globally (1). TB has been termed an infectious disease of poverty (IDoP) (2). Conceptualizing TB as an IDoP is useful when considering the vulnerable populations that have historically incurred a disproportionate TB burden. The Centers for Disease Control and Prevention (CDC) indicates that there is a disproportionate TB disease burden among minority and foreign-born populations in the U.S. (3). Along the U.S./Mexico border, the unequal TB disease burden pattern among poor, minorities, and foreign-born populations holds true (Table 1).

**Table 1:** **Tuberculosis incidence rates (cases per 100,000)**.

	Yuma	Arizona	U.S.
2009	10.3	3.5	3.8
2008	15.2	3.5	4.2
2007	12.4	4.7	4.4
2006	26.6	5.0	4.6

*Arizona Department of Health Tuberculosis Surveillance Report, 2010*.

Yuma County is located on the Arizona/Mexico border. In 2015, Yuma County reported an incidence rate of 9 per 100,000, which greatly exceeds the Arizona state TB incidence rate of 1.1 cases per 100,000 people in 2015 (4). In Arizona, 57.1% (*n* = 113) of TB cases were among individuals aged 25–64 years of age for 2015 (4). Males in Arizona accounted for 70% (*n* = 139) of active TB cases in Arizona in 2015 (4). This data trend is consistent with epidemiological trends reported in previous years (5).

Mexico’s aggregate country level data suggest a TB burden rate of 21 per 100,000 people in 2015 (6). In 2010, the highest TB incidence rates for Mexico were reported in the Northern border states (7). Sonora is the Mexican Northern border state adjacent to the state of Arizona. The TB incidence rate for 2010 in Sonora was reported as 32.9 cases per 100,000 (7). Sonora’s TB incidence rate exceeds the national average by 11.9 cases per 100,000. In 2015, Arizona reported that 35.4% (*n* = 70) of the TB cases found in the state were among people born in Mexico (4).

Successful TB treatment completion persists as a substantial challenge. The World Bank estimates that 80.0% of new TB cases completed treatment in Mexico in 2014 (6). Arizona reports a comparable TB treatment completion rate of 86.3% in 2015 (4). The rigors of TB treatment have been demonstrated to complicate seeking medical attention and treatment adherence particularly among individuals with a high degree of familism, which is a Mexican cultural expectation (8). Qualitative data collected at the U.S./Mexico border indicates TB treatment resulted in feelings of isolation from loved ones, ostracization from community members, loneliness, and conceptualization of “filthiness” (9). Identifying TB treatment predictor variables for border populations is imperative for increasing the completion rate along the U.S./Mexico border. A concrete understanding of the mechanisms, which encourage or disallows TB treatment completion provides a framework for the development of public health intervention strategies.

## Methods

Retrospective medical chart (*n* = 439) reviews was performed on all patients treated for TB at Yuma County Health Department (YCHD) (*n* = 160) and Centro de Salud San Luis Río Colorado (CDS San Luis) (*n* = 279) between January 1, 2005 and December 31, 2009. All medical charts provided by the clinical facilities were de-identified. The investigators were not privy to any identifying information for the patient included in the study. Complete chart information for CDS San Luis was limited to information reported to the state’s public health district office located in Caborca, Sonora, Mexico. Analysis focused on de-identified data provided by both health departments. Internal Review Board (IRB) approval was not required for this study. The quantitative analysis was conducted using the statistical software package STATA/SE 11.0.

Patients undergoing treatment were excluded from analysis as completion or abandonment status could not be determined for this segment of the study sample. Four treatment outcomes were identified: 1. completed; 2. abandoned; 3. moved/deported; 4. death (Table 2). Patient charts with treatment outcome of moved/deported or death were excluded from the analysis. The data were handled as dichotomous categories with the outcome variable being treatment completed or treatment not completed. Individuals who stopped treatment regardless of the reason were categorized as treatment not complete for all of the analyses conducted in this study. A total of 131 patients (*n* = 131) were categorized as did not complete treatment. Following exclusion of patients who did not complete treatment, the sample size used for further analysis was 308 (*n* = 308).

**Table 2:** **Treatment outcomes: completed, abandoned, moved/deported, died**.

Outcome	Yuma (*n* = 160)	CDS San Luis (*n* = 279)	All cases (*n* = 439)
Completed	71.25% (114/160)	69.53% (194/279)	70.16% (308/439)
Abandoned	4.38% (7/160)	13.98% (39/279)	10.48% (46/439)
Moved/deported	20.00% (32/160)	11.47% (32/279)	14.58% (64/439)
Died	4.38% (7/160)	5.02% (14/279)	4.78% (21/439)

Data extracted from chart reviews included treatment location (YCHD or CDS San Luis), patient age at diagnosis, gender, ethnicity, country of origin, date of arrival in the U.S., and whether patient frequently crosses the U.S./Mexico border. Descriptive statistics for the study sample were captured with proportions and chi-square testing with significance defined at the *p* < 0.05 level to determine differences between the populations receiving treatment at YCHD versus CDS San Luis (Table 4).

Following the analyses to generate the descriptive statistics, the data (*n* = 308) were dichotomized as completed treatment and abandoned treatment (Table 3). The dichotomized data were reviewed and analyzed using chi-square, unadjusted odds ratios, and logistic regression to identify predictor variables for TB treatment completion. Bivariate analyses were conducted to examine the association between potential predictors and treatment completion using chi-square testing and determining *p* values for statistical significance, unadjusted odds ratios, and 95% confidence intervals. Associative relationships trending toward significance at the *p* < 0.1 level were evaluated because of the small sample size.

**Table 3:** **Dichotomous treatment outcome (completed versus did not complete)**.

	Yuma (*n* = 160)	CDS San Luis (*n* = 279)	All cases (*n* = 439)
Completed	71.25% (114/160)	69.53% (194/279)	70.16% (308/439)
Did not complete	28.75% (46/160)	30.47% (85/279)	29.84% (131/439)

The final analysis was the assessing of the data *via* logistic regression. Logistic regression models for combined site data and separate site data were created to determine best models of treatment completion. Logistic regression modeling was performed inserting all potential predictors and sequentially eliminating the variables found to be statistically insignificant at the value of significance being *p* = < 0.1. The potential predictors were then inserted and deleted in a stepwise fashion allowing the predictors demonstrating significance or trending toward significance (*p* = < 0.1) to be included in the model.

## Findings

Male patients constituted the majority of the sample (*n* = 327), which echoes overall TB epidemiological trends of higher TB rates among males. A larger gap in the number of males being treated at each respective facility was seen with 105 male patients at YCHD and 222 male patients at CDS San Luis. YCHD treated a significantly greater percentage of female patients (34.4%, *X*^2^ = 10.41, *p* = 0.001).

Patient ages for all charts reviewed ranged from 3 months to 90 years with a combined average age of 37.4 years. The average age of patients treated through YCHD was 35.9 years. For patients treated through CDS San Luis, the average age was 38.3 years. Patients at YCHD were found to be younger than 26 years of age at a statistically significant rate (36.2%, *X*^2^ = 18.42, *p* < 0.001).

Patients being treated at CDS San Luis were more likely to be cases referred by a facility such as jail or rehabilitation center (29%, *X*^2^ = 9.01, *p* = 0.003). CDS San Luis patients were also significantly more to report drug use (46.6%, *X*^2^ = 48.5, *p* < 0.001) (Tables 6 and 7).

CDS San Luis treated a significantly larger percentage of new smear positive cases (64.9%, *X*^2^ = 15.27, *p* < 0.001). There was not a statistically significant difference in treatment of individuals who had not previously been diagnosed across the two facilities (*X*^2^ = 2.12, *p* = 0.16) (Table 5).

Patients treated for TB at YCHD were more likely to complete treatment than patients treated at CDS San Luis [OR 3.27, 95% CI (1.44, 7.40), *p* = 0.004]. Twenty percent (20%) of YCHD patients were lost to follow-up due to relocating outside of the YCHD jurisdiction. Patients treated at YCHD completed treatment at a rate of 71.25% (114/160) versus a 69.53% (194/279) patients completion rate at CDS (Tables 2 and 3).

Being female was identified as a predictor for successful treatment completion [OR 3.71, 95% CI (1.46, 9.38), *p* = 0.004] (Tables 5 and 6). Individuals in all other age categories except the 36–45 years of age were more likely to complete treatment [OR 2.42, 95% CI (1.26, 4.66), *p* = 0.007]. First diagnosis of TB [OR 2.82, 95% CI (1.44, 5.52), *p* = 0.002] and first treatment course for TB [OR 3.03, 95% CI (1.54, 5.95), *p* = 0.001] were both found to be predictors for successful completion of treatment. No reported drug use was a predictor for treatment completion [OR 3.05, 95% CI (1.63, 5.70), *p* = 0.0003]. HIV negative status was also identified as a predictor for successful treatment completion [OR 11.07, 95% CI (0.84, 145.63), *p* < 0.1]. For all cases, no reported excess alcohol use trended toward significance as a predictor of treatment completion [OR 1.88 (0.95, 3.73), *p* = 0.07] (Table 4).

**Table 4:** **Factors associated with treatment completion (unadjusted odds ratios)**.

	Yuma cases (*n* = 114)	Yuma OR 95% CI *P*-value	CDS San Luis cases (*n* = 194)	CDS San Luis OR 95% CI *p*-value	All cases (*n* = 308)	All cases OR 95% CI *p*-value
Treated through Yuma County Health Department or CDS San Luis	71.25% (114/160)		69.53% (194/279)		70.16% (308/439)	OR = 3.27 (1.44, 7.40) *p* = 0.004**
Female	95.92% (47/49)	1.75 (0.37, –), *p* = 0.51	94.23% (49/52)	OR = 4.06 (1.27, 12.9) *p* = 0.02**	95.05% (96/101)	3.71 (1.46, 9.38) *p* = 0.004**
Pansensitive case	97.77% (44/45)	8.0 (0.94, –) *p* = 0.06*		N/A		N/A
Non-institutionalized	93.24% (69/74)	0.61 (0.00, 2.8) *p* = 0.56	86.71% (137/158)	2.06 (1.03, 4.12) *p* = 0.04**	88.79% (206/232)	1.55 (0.83, 2.90) *p* = 0.17
Not 36–45 years	94.12% (98/102)	0.89 (0, 6.11) *p* = 0.92	86.86% (152/175)	2.52 (1.23, 5.15) *p* = 0.01**	89.53% (248/277)	2.42 (1.26, 4.66) *p* = 0.007**
Diagnosed other than in year 2005	94.74% (90/95)	1.5 (0, 7.22) *p* = 0.64	86.02% (160/186)	2.35 (1.11, 5.00) *p* = 0.02**	88.97% (250/281)	2.09 (1.07, 4.08) *p* = 0.03**
Diagnosed in 2008	95.00% (19/20)	1.2 (0.18, –) *p* = 0.87	95.0% (38/40)	4.51 (1.15, –) *p* = 0.03**	95.0% (57/60)	3.25 (1.03, 10.20) *p* = 0.04**
No excess alcohol use	95.92% (94/98)	3.52 (0.82, 15.4) *p* = 0.098*	84.78% (156/184)	1.61 (0.75, 3.49) *p* = 0.23	88.65% (250/282)	1.88 (0.95, 3.73) *p* = 0.07*
No drug use	95.50% (106/111)	5.3 (0, 28.44), *p* = 0.04**	88.00% (110/125)	2.10 (1.04, 4.20) *p* = 0.04**	91.52% (216/236)	3.05 (1.63, 5.70) *p* = 0.0003**
Not HIV positive	94.91% (112/118)	9.33 (0, 85.24) *p* = 0.04**	83.84% (192/229)	5.19 (0.88, 30.43) *p* = 0.07*	87.61% (304/347)	5.30 (1.28, 21.99) *p* = 0.02**
First tuberculosis treatment	95.28% (101/106)	3.37 (0, 17.14) *p* = 0.15	86.49% (160/185)	2.64 (1.26, 5.54), *p* = 0.01**	89.69% (261/291)	3.03 (1.54, 5.95) *p* = 0.001**
Smear negative	97.37% (37/38)	2.58 (0.35, –) *p* = 0.40	93.33% (42/45)	3.36 (1.04, 10.7) *p* = 0.04**	95.18% (79/83)	3.99 (1.44, 11.05) *p* = 0.006**
Not binational case	95.41% (104/109)	4.16 (0, 21.63) *p* = 0.09*	83.00% (190/229)	N/A	86.99% (294/338)	0.95 (0, 3.91) *p* = 0.95

*p < 0.1.

**p < 0.05.

**Table 5:** **Model predicting treatment completion: all cases (*R*^2^ = 0.2116, *n* = 308)**.

Predicts completion	*n*	Odds ratio (95% confidence interval)
Diagnosed in 2008 (index 2005)	81	7.89 (1.82, 34.11)**
Diagnosed in 2009 (index 2005)	85	5.02 (1.54, 16.34)**
Female	112	4.90 (1.29, 18.60)**
Location of treatment Yuma	160	3.98 (1.17, 13.52)**
No prior tuberculosis diagnosis	228	3.61 (1.63, 7.98)**
Age >45 years (index 26–45 years)	137	3.13 (1.22, 8.02)**
Smear negative case	111	3.00 (0.94, 9.56)*

*p < 0.1.

**p < 0.05.

**Table 6:** **Model predicting treatment completion: CDS San Luis cases (*R*^2^ = 0.2082, *n* = 194)**.

Predicts completion	*n*	Odds ratio (95% confidence interval)
Diagnosed in 2008 (index 2005)	50	12.52 (2.28, 68.89)**
Smear negative case	59	5.58 (1.23, 25.24)**
Age >45 years (index 36–45 years)	79	5.45 (1.68, 17.66)**
No prior tuberculosis diagnosis	138	4.36 (1.80, 10.56)**
Female	57	4.11 (1.04, 16.24)**
Age <26 years (index 36–45 years)	50	3.88 (1.06, 14.17)**
Diagnosed in 2009 (index 2005)	61	3.52 (1.07, 11.53)**
Not HIV positive	192	11.07 (0.84, 145.63)*
Age 26–35 years (index 36–45 years)	78	2.24 (0.88, 5.73)*

*p < 0.1.

**p < 0.05.

**Table 7:** **Model predicting treatment completion: Yuma cases (*R*^2^ = 0.1017, *n* = 114)**.

Predicts completion	*n*	Odds ratio (95% confidence interval)
No drug use	106	6.5 (1.03, 41.07)**
Not HIV positive	112	13.00 (0.96, 175.01)*

*p < 0.1.

**p < 0.05.

## Limitations

Patient mobility proved to be a challenge for this study. Patient relocation placed substantial constraints on the analysis. The large number of participants lost to follow-up reduced the overall sample size. Future research should seek to establish further predictor variables in a sample located in a similar binational setting. Future research should also consider utilizing a mixed methods approach to identify modifiers of treatment completion that would not be captured in medical chart data.

## Discussion

High mobility of the patient population is a key element of this study. The rate of mobility provides an insightful commentary of the population and the geographic location of this study. It is unclear if participants continued treatment following relocation. The movement of patients with active TB is a concern for public health. High mobility is not unique to the Yuma/San Luis area. High mobility is a phenomenon seen across the U.S./Mexico border. In an attempt to address this border reality, the Arizona Department of Health Services TB Control Program (ADHS) utilizes various strategies to achieve continuity of care for active TB patients. One strategy employed by ADHS are “Meet and Greets,” which are coordinated by ADHS, the Office of Border Health, and the health department in Mexico (10). Meet and Greets use the San Luis port of entry for cases returning to Mexico. The objective of Meet and Greet program is to provide medical case management to encourage treatment continuation despite the need for relocation. Over 2013, there were a total of five Meet and Greets (10). The expansion of Meet and Greets has the potential for minimizing TB treatment abandonment because of patient relocation. Further research on the contribution of Meet and Greets on successful TB treatment completion would be useful for better understanding mechanisms to address infectious diseases among mobile populations.

Tuberculosis diagnosis in San Luis presents an additional structural barrier faced by the study sample. The necessary technology for TB diagnosis is not available at the CDS San Luis facility. YCHS does have the technology and capacity to test for TB, however, non-U.S. citizens are ineligible to utilize facility services. For CDS San Luis patients, the nearest facility where they have access to TB diagnosis technology is in Hermosillo, Sonora, Mexico. Hermosillo is approximately a 6-h drive from the patient population of San Luis. The economic costs incurred by the patient to access services in Hermosillo for TB testing is a huge deterrent. For many of the CDS San Luis patient population finding, the time and resources in order to make the trip to Hermosillo is an impossibility. Allowing San Luis residents to access YCHS TB detection services would be a positive contribution to the public health situation of the geographic region.

The findings of this study indicate that Mexican males aged 36–45 years of age were more susceptible for acquiring TB and were simultaneously least likely to complete TB treatment. Previous findings have asserted that economic forces dictate treatment completion among Mexican populations. As both clinical sites are public health facilities, which provide services either free or on an income-based sliding scale. Economic difficulties resulting from TB disease burden in this binational context has substantial implications. TB can prove to be economically devastating at the individual, household, and community levels (11). Males aged 18–64 years are in their working prime and were disproportionately infected with TB. The TB disease burden for these males renders them unable to actively labor and produce. For men, the perceived potential of diminished economic opportunities is an established mechanism that discourages seeking medical care for TB (12). A high TB burden stifles economic indicators when a key segment of the country’s work force is incapacitated by a debilitating physical illness. Additionally, family members, most often women, involved in providing care for the ill individual are unable to contribute to the economy *via* full time employment. Retaining gainful employment has been identified as taking priority over TB treatment for a patient population in the Mexican state of Chiapas (13).

Patients treated through CDS San Luis were more likely to have reported drug use (46.6%) than patients treated through YCHD (13.8%). The medical charts provide few insights as to why a 32.8% difference in reported drug use would be seen across the two clinical site populations. The study findings indicated that reported drug use was associated with the individual being less likely to complete their TB treatment. A potential explanation for the large difference of reported drug use across the two clinic sites could be a fear of deportation for engaging in an illegal activity. As the findings show the U.S. clinic has a smaller proportion of the study sample, reporting use of drugs would be a fear of reporting an illegal activity resulting in deportation. Fear of deportation for the TB patient, family members, or an individual in their social network has been asserted as a reason individuals feel a reluctance to seek treatment for TB (14). Perceived fears of deportation is of particular importance for our discussion because of the binational setting of this study. Stigma produces discrimination (9), thereby minority or foreign-born status individuals with TB face a double burden of racial discrimination and TB stigma-associated discrimination. The fear of deportation in a binational setting is a formidable force across multiple health indicators.

An HIV negative status was associated with successful TB treatment completion. TB stigma persists as a contemporary impediment for TB detection and treatment. A similar stigma is associated with a HIV-positive diagnosis. Health-related stigma is characterized by the individual’s perceptions of potential for exclusion, blame, devaluation, and reasonable expectations of adverse social judgments, which results in a deterrent for seeking medical care or treatment adherence (15). TB risk factors often intersect with other forms of social stigma such as incarceration, homelessness, or co-occurring HIV/AIDS (16). TB stigma ties to processes at the institutional, community, and interpersonal level (12). Co-infection of TB and HIV has been deemed as one of the biggest challenges for public health and clinical treatment of HIV patients globally (17).

Contextualizing TB as a social disease provides an opportunity to evaluate the complex social forces that inform the epidemiological disease trends moving away from an individual level behavioral driven model of understanding. An unequal distribution of TB disease burden among traditionally marginalized populations demonstrates the role of social disadvantage as they relate to health outcomes at the individual level (18). The study findings confirm previous studies regarding TB disease patterns constructed by cultural, social, and institutional forces (19). Findings from this study identify mechanisms, which influences successful completion of TB treatment in a binational context. These findings demonstrate the need for socio-ecological-based interventions to curtail TB as an endemic disease outcome along the U.S./Mexico border.

## Ethics Statement

The study was a secondary data analysis of de-identified medical charts, which does not require IRB approval.

## Author Contributions

The authors have made the following contributions to the submitted manuscript: substantial contributions to design of the work, analysis, and interpretation of data for the work (CV, KE, and CR); drafting the work (CV) and reviewing it critically for important intellectual content (KE and CR); final approval of the version to be published, agreement to be accountable for all aspects of the work in ensuring that questions related to accuracy or integrity of any part of the work are appropriately investigated and resolved (CV, KE, and CR).

## Conflict of Interest Statement

The authors declare that the research was conducted in the absence of any commercial or financial relationships that could be construed as a potential conflict of interest. The reviewer, JG-G, and the handling editor declared their shared affiliation, and the handling editor states that the process nevertheless met the standards of a fair and objective review.
